# The Profession and Government "Blacklegs"

**Published:** 1913-09-20

**Authors:** 


### THE NATIONAL MEDICAL GUILD.

President: CHAS. BUTTAR, M.A., M.D., B.C.

Gen. Sec.: P. C. RAIMENT, M.R.C.S., L.R.C.P.

Offices: 34 Villiers Street, Strand, London, W.C.

### THE PROFESSION AND GOVERNMENT "BLACKLEGS."

We wrote last week of the sorrow and pain that one felt at the action of the medical men of Blackpool in the face of the threats of the Commissioners, and we wish to return to the subject as we feel that the whole future of the profession lies in resisting these attacks.

One almost feels tempted at times to despair at the prospect that faces the profession unless they are prepared to take their courage in both hands and defy the enemy, and unless we can strengthen these weak links, then is our whole chain in vain; so in the hopes of stiffening the weaker brethren a little we venture to offer them certain theorems to think over. If we can succeed in proving the truth of these and they will be led to believe them, we think that they will pause and consider their future actions a little more carefully.

In the first place, will they admit that we, the medical men of this generation, are the custodians of the profession of medicine, and that it is our moral and bounden duty to hand on the heritage that we have received in an unsullied condition? It is a common mistake for a generation to think that they are an entity in themselves and to forget that they owe much to their predecessors, and that their successors will look to them to discharge the debt that they owe to the time before them. We know how our forerunners in the profession of medicine have raised the status from little better than that of a tradesman to the proud position that we occupied at the commencement of this century. Can this generation say that the trust thus committed to them has been faithfully fulfilled? Can we say that we hold such a high position in the eyes of the nation as we held, say, this time last year? If not, to whom will the blame be apportioned when the historian of the future deals with the matter? Surely to those who put their material welfare before the honour of the profession and the advancement of the science and art of medicine. And such a little was needed to stay the tide! Here in London the exhibition of a little courage and a little fight held the majority of the practitioners off the panel for three months at least, and if only a similar state of things had taken place in the country could the Government have stood out for that length of time?

### The Threat of Whole-time Doctors.

The second theorem that we have to advance is that the threatened employment of whole-time medical officers is a bogey that is only produced when the profession ceases to be amenable to the wishes of the Commissioners. Let us consider this a little more in detail. It will be well within the recollection of everyone that in January last, when the profession was being bludgeoned to heel by the emissaries of the Government, it was freely stated that they proposed to set up a whole-time service in every town where the profession proved recalcitrant. The impossibility of this proposition should be apparent to everyone.

The Medical Register is limited in numbers, and the number of unattached medical men is very small, as everyone who has required a "locum" this summer will speedily testify. It is not to be doubted that in any particular town such an establishment would be possible, but to assume that such a service could be set up everywhere was one of those incredible pieces of folly of which the profession was guilty at that time. The number of whole-time "blacklegs" at the disposal of the Government was stated on good authority to be about fifty, though it might well have been more, but that it was a negligible quantity is shown by their action in London. Here with a panel list woefully attenuated, despite their most awful threats, very few boroughs have ever seen the "whole-timer," and in those places in which he has managed to obtain a footing by this time he has in many cases left owing to his inability to charm away the patients of the local practitioners. So much for the whole-time bogey!

### The Treatment of Temporary Residents.

The above is only a matter of history, but the lesson learnt last January has apparently not sunk deep enough to prevent the profession repeating the follies of that disastrous month. To apply the reasoning to the unfortunate accident at Blackpool last month:

We believe that Blackpool is not alone in its rejection of the terms of the Commissioners for temporary residents, although on that point it is difficult to obtain accurate and trustworthy information, but we may assume that a fair quantity of the health resorts feel the same way about it. That being so, where do the Government propose to obtain a sufficient supply of medical men to police the health resorts of England. To anyone who has tried in vain to obtain "locums" this year the proposition is unthinkable. Of course they could, if they wished, break up the resistance in one town, but that is where our organisations should step in, and it is the bounden duty of every member of the profession to subscribe and raise such funds that those men shall not be injured in their endeavour to uphold what they believe to be the best interests of the profession. The injury that they would receive does not alarm us very much. The treatment of temporary residents does not represent a very magnificent income even if the whole be shared by one or two persons, and the support that they are likely to receive locally does not, if past experience be worth anything, promise to be very great.

We do most heartily commend to the notice of the profession the above arguments, because if they are properly understood the Government ought to have the greatest difficulty in resurrecting their old bogey, whose ashes we fondly imagined had received decent burial some time ago.

### The Limit of Endurance.

And that takes us a step farther. This treatment of temporary residents is a small point compared with the principle involved. Here we have the local profession united in their belief that the proposals of the Commissioners are unfair to them, and yet they give way, and that before a shot is fired or a man killed. Where is it going to stop? Is there any point at which the profession will refuse unanimously to have anything to do with it? There must be, but we have not yet reached it. Are we going through years of miserable skirmishing, always with one result, before we find a platform on which we can make a stand? Because that is the way the profession is drifting unless it can once for all make up its mind to cry a halt and to engage the pursuing army in a pitched battle.

It is the objection of many people that the trade union stands for a strike, and it is quite a legitimate and valid reason for refusing to attach one's name to the trade-union movement, but to those people we wish to put this question. "Bearing in mind the methods that the Commissioners have displayed both in getting their machine to work and in keeping it working, is there any method left to us by which we can retain the foothold that we have lost, other than by a resolute and dignified refusal to accept the terms offered?" If the answer is in the affirmative, then we have succeeded in converting an individualist into a trade unionist, and taught him that a strike is not a matter of pickets, brass bands, and brickbats, but simply a refusal of the terms and a determination to hold to that refusal at all costs, and further to help others to keep their word.

Not until we can arrive at that stage can we hope to come back again into our own, nor until we have learnt the bitter lesson by a series of reverses, petty in character but humiliating in spirit, can we hope to see the profession once again united and doing its work in a willing and contented frame of mid.

